# Pioneers of Origin of Life Studies—Darwin, Oparin, Haldane, Miller, Oró—And the Oldest Known Records of Life

**DOI:** 10.3390/life14101345

**Published:** 2024-10-21

**Authors:** J. William Schopf

**Affiliations:** Department of Earth, Planetary, and Space Sciences, University of California, Los Angeles, CA 90024, USA; schopf@epss.ucla.edu; Tel.: +310-825-1170

**Keywords:** abiotic synthesis, adenine synthesis, Apex chert, dawn animal of Canada, Gunflint chert, Miller–Urey synthesis, Paleoarchean, Precambrian, primordial soup, Strelley Pool Formation

## Abstract

The two basic approaches to elucidating how life began both date from Darwin. The first, that of the experimentalists, stems from Darwin’s famous “warm little pond” letter to Joseph Hooker of 1871. This approach, an attempt to replicate the sequential events leading to life’s origin, is exemplified by the “primordial soup” hypothesis of A.I. Oparin (1924) and J.B.S. Haldane (1929); the Miller–Urey laboratory synthesis of amino acids under possible primitive Earth conditions (1953); and Joan Oró’s nonbiological synthesis of the nucleic acid adenine (1959). The second approach, that of the observationalists who search for relevant evidence in the geological record, dates from Darwin’s 1859 *On the Origin of Species*, in which he laments the “*inexplicable*” absence of a pre-Cambrian fossil record. Darwin’s concern spurred a century of search that was ultimately rewarded by Stanley Tyler’s 1953 discovery of diverse microscopic fossils in the ~1900 Ma Gunflint Chert of southern Canada. Tyler’s find was soon followed by a cascade of discoveries worldwide; the establishment of a new field of science, Precambrian paleobiology; and, more recently, the discovery of 3400 and ~3465 Ma Paleoarchean microfossils, establishing that primordial life evolved early, far, and fast. Though progress has been made, much remains to be learned in the foci of this *Origin of Life 2024* volume, for which this essay is the history-reviewing “stage setter”.

## 1. Darwin States the Problem

**Figure 1:**
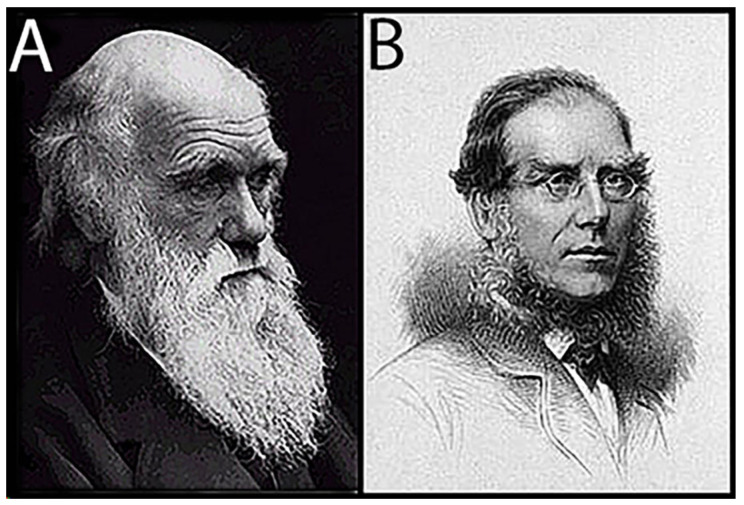
(**A**) Charles Robert Darwin. (**B**) Joseph Dalton Hooker.

Darwin (1809–1882; Figure 1A) was flummoxed! As he wrote in *On the Origin of Species* [1] (Chapter X): “If the theory [of evolution] be true, it is indisputable that before the lowest Cambrian stratum was deposited … the world swarmed with living creatures. [Yet] why we do not find rich fossiliferous deposits belonging to these assumed earliest periods … I can give no satisfactory answer. The case at present must remain inexplicable; and may be truly urged as a valid argument against the views here entertained”. Indeed, sed as an oft-repeated “argument against” Darwin’s ground-breaking theory, it certainly was, as I discovered in the mid-1960s when my reports of the discovery of Precambrian fossils elicited an onslaught of postcards and letters from religious zealots literally “damning me to Hell”—and worse. After all, these strident Bible thumpers opined, Darwin doubted his own theory, so who was I to have the arrogance, the sheer gall to claim that his doubt was misplaced?

Darwin’s opus focused on life’s evolution, the seemingly ever-changing march of life through time spurred by “natural selection”, not on life’s beginnings. Indeed, he regarded the origin of life as being essentially unfathomable, a view clearly expressed in a letter of 1863 to his botanist friend Joseph. D. Hooker (1817–1911; Figure 1B) in which he wrote “*It is mere rubbish thinking, at present, of origin of life; one might as well think of origin of matter*”. In 1868, in his volume on *The Variation of Animals and Plants under Domestication* [2], he reiterated this view, stating that life’s origin was “*quite beyond the scope of science*”. Then, in 1871, Darwin wrote a now famous letter to Hooker that included his speculations on how life began: *“It is often said that all the conditions for the first production of a living organism are now present, which could ever have been present. But if (and oh what a big if) we could conceive in some warm little pond with all sorts of ammonia and phosphoric salts,—light, heat, electricity &c. present, that a protein compound was chemically formed, ready to undergo still more complex changes…”* But this could never happen now since: *“… at the present day such matter wd be instantly devoured, or absorbed, which would not have been the case before living creatures were formed”.* As is well evidenced by the contributions included in this present volume, *Origin of Life 2024*, Darwin’s “warm little pond” has itself evolved over the past century and a half, as the solution to life’s origin—arguably the greatest unsolved mystery in modern science—has increasingly come into focus.

## 2. Oparin Suggests a Solution

**Figure 2:**
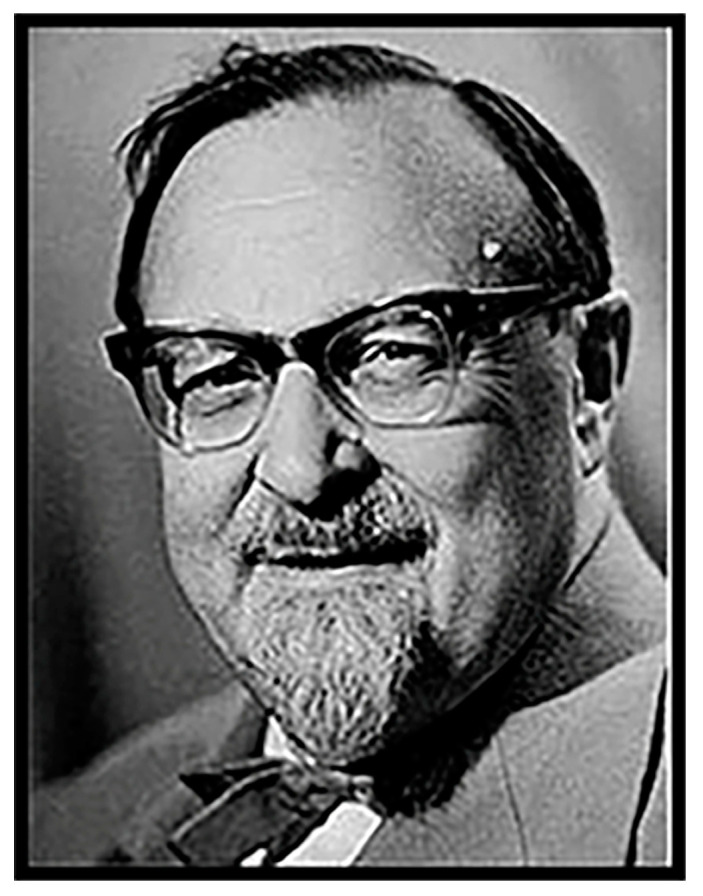
Aleksandr Ivanovich Oparin.

**Figure 3:**
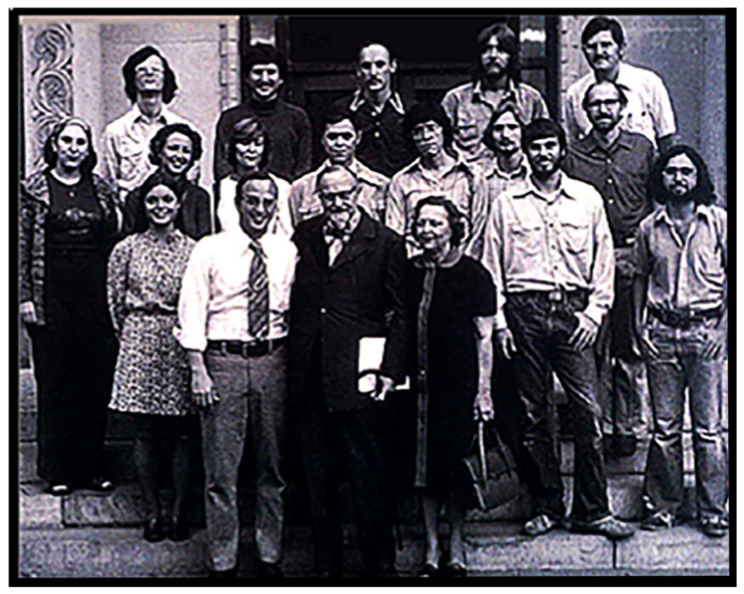
Front, left to right: J. William Schopf, Aleksandr I. Oparin, and his Russian translator in 1976, with their UCLA class behind.

The initial, pivotal impetus that led to this progress was provided in 1924 by Aleksandr I. Oparin (1894–1980; Figure 2). I first met Oparin in 1974 when he was President of ISSOL, The International Society for the Study of the Origin of Life (known also as the “The International Astrobiology Society—ISSOL”). We became good friends in 1975 when I was a US-USSR National Academies of Sciences Exchange Scientist and spent eight weeks of my six-month visit at the A.N. Bach Institute of Biochemistry in Moscow, which he headed (and which, in 1995, elected me as the first Honorary Foreign Member of its Scientific Council, reputedly the first “secret ballot” in the history of the Institute). In 1976, following my return to UCLA, Oparin spent two months as a visiting professor in my department (Figure 3), where he delighted in referring to me as his “American grandson”. Later, in 1989, I was awarded ISSOL’s A.I. Oparin Medal, which would have pleased him even more.

**Figure 4:**
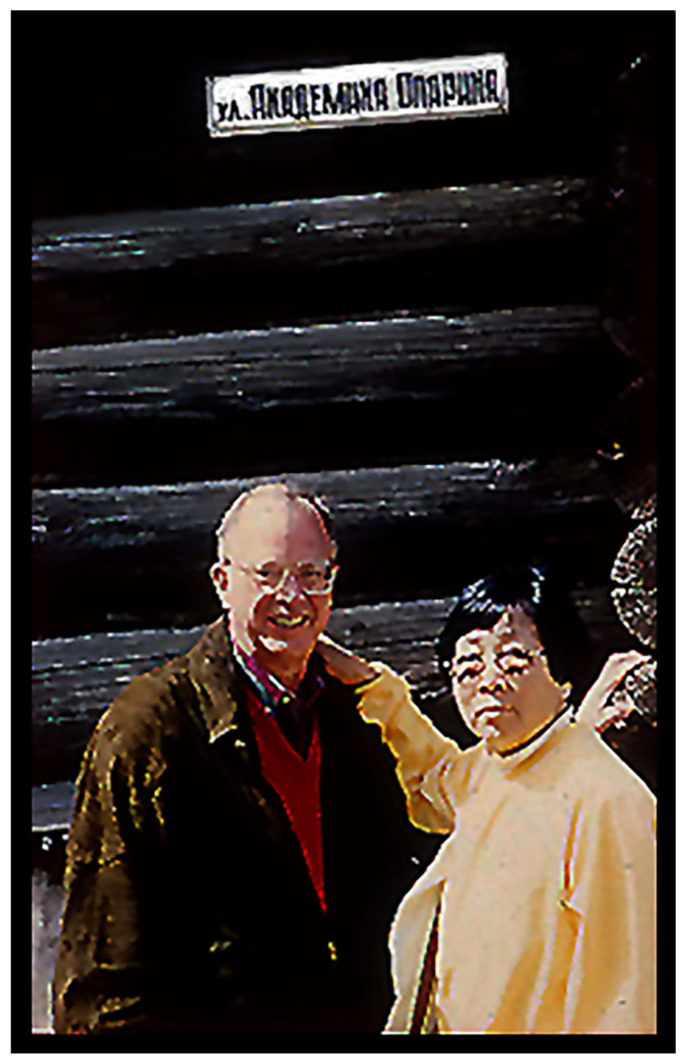
J. William Schopf and wife Jane Shen Schopf in front of the street sign for “Academician Oparin Street”, Uglitch, Russia (May 2006).

Oparin was born in Uglitch, northeast of Moscow on the Volga River (where a major street is now named in his honor; Figure 4) and was brought up in the nearby village of Kokayevo. In 1912, upon his graduation from high school, Oparin elected to take the All-Russian National Examination (the equivalent of the USA’s Scholastic Aptitude Test) to determine whether he was qualified to enter college. He and one of his classmates performed excellently, so well, in fact, that they were admitted to Moscow State University (MSU), the top-ranked university in the country. Their science teacher arranged to take them to the university so they could see what lay in store before they enrolled.

**Figure 5:**
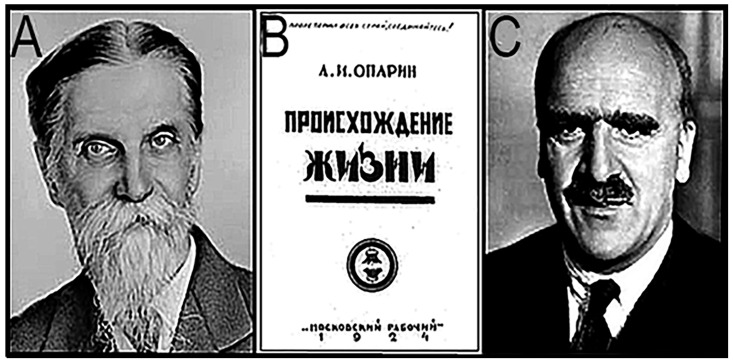
(**A**) Klemit Arkadievich Timiryazev. (**B**) Oparin’s 1924 book, *The Origin of Life* (in Cyrillic, *Прoисхoждение жизни*). (**C**) John Burdon Sanderson Haldane.

As fate would have it, they missed the morning train, and when they finally arrived at the university in the mid-afternoon, there was time available to attend only a single class. Oparin chose the botany class of Klemit A. Timiryazev (1843–1920; Figure 5A), primarily because he had read Timiryazev’s textbooks in high school and, being interested in botany, had used them to design some simple plant-growth experiments. As it turned out, his selection of this class was a game-changer, both for Oparin and for the origin of life studies.

Timiryazev was a widely known botanist and scholar, acclaimed for his enthusiastic oratory, and was one of only a few outspoken proponents of Darwinian evolution in all of theocratic Czarist Russia. Moreover, he was about to retire from the professoriate, so Oparin was to hear what in essence was Timiryazev’s farewell address.

As Oparin recounted to me, Timiryazev focused on his life history, most notably on how, as a 27-year-old recent Ph.D., he had visited Darwin at Down House in the rolling hills of Kent, on the outskirts of London, and strolled with Darwin along the pebbled “sandwalk” at the back of the property where he learned the basics of evolution from the Master, became convinced of the validity of Darwin’s theory, and thought to himself: “If Darwin can do this for animals, I can do it for plants!” which, with considerable success, he went on to do. Oparin was enthralled. But as he later thought through the matter, he discovered a glaring flaw. As Oparin phrased it to me, *“Darwin had revealed the evolution of animals, and Timiryazev showed the evolution of plants, but no one had written the first chapter of the book, how life originated”.*

Almost immediately, Oparin began to work on the problem, and by 1920, three years after his graduation from MSU, he had completed a manuscript that he submitted for publication. The paper was rejected, according to Oparin, by the Czar’s censors, who had remained in place even as the 1917–1923 Bolshevik Revolution raged on. Surprisingly, perhaps, Oparin viewed this setback as “*the best thing that could have happened since it allowed* [him] *to bring the manuscript up-to-date by including new estimates of the composition of the primordial atmosphere”.* In particular, the time lag had allowed Oparin to take into account the then-recent discovery of methane in the atmospheres of Jupiter and the other giant planets, leading him to suggest that the primordial Earth possessed a strongly reducing atmosphere containing methane, ammonia, hydrogen gas, and water vapor, the mixture he proposed as the starting materials for the beginnings of life.

His updated, revised version, *The Origin of Life* [3], Figure 5B, was published in 1924, shortly after the end of the Revolution. However, written in Russian, it elicited little interest among non-Russian speaking scholars, and, given that it appeared directly following the Marxist–Leninist takeover of Czarist Russia—and presented a naturalistic, rather than theistic, explanation of life’s beginnings that fit well with Marxist dialectical materialism—the scenario it presented was regarded as politically suspect in the West.

Thus, in some quarters, Oparin was viewed as an untrustworthy “Godless Commie”, an assumption seemingly reinforced by his later public support of Trofim Lysenko (1898–1976) whose shoddy research promoting the non-genetic inheritance of acquired characteristics destroyed large swaths of Soviet agriculture and served as fodder for Communist Party General Secretary Joseph Stalin’s (1878–1953) goal of “perfecting” modern Soviet citizens. Oparin’s rather widely assumed guilt by association, however, was unwarranted. Given Oparin’s towering national scientific reputation, it would have been odd were he not to have met Stalin, but he rarely talked of politics and was never a member of the Communist Party. Moreover, as he explained to me, his support of Lysenko “*was based on* [their] *personal friendship, not on politics, not on science*; [they} *had adjoining dachas on the outskirts of Moscow where* [their] *families would meet, relax and share dinners during the summer holidays and* [their] *children would play together*”.

## 3. Haldane Suggests the Same Solution

Rather remarkably, in 1929, only five years after the publication of Oparin’s book, the British Indian evolutionary biologist J.B.S. Haldane (1892–1964; Figure 5C), a professor at the University College London, independently came up with virtually the same ideas as those proposed by Oparin—which, because they were published in English, were far more accessible to non-Russian speaking scientists. His contribution was brief, an eight-page article in *The Rationalist Annual* [4]. But because Haldane was an outspoken card-carrying member of Great Britain’s Communist Party, the “naturalistic notions” he presented were regarded as politically motivated and were largely rejected by the scientific community. Indeed, it was not until 1938 when the Macmillan company published an annotated English translation [5] of Oparin’s originally short, 71-page book—which he referred to affectionately as his “little pamphlet”—that the Oparin–Haldane “primordial soup” hypothesis began to gain traction.

It is important to note that it was not solely international politics that held up progress. Rather, a major role was played by Oparin’s notion that “*sound theory can stand alone … the facts will follow*”. Thus, his “little pamphlet”, much like Haldane’s even shorter essay, is idea-rich but devoid of supporting experimental data. Although at present this may seem odd, in the 1920s this so-called “Primacy of Theory” concept found a receptive audience in Russia, just as it does today in some developing countries, where until recently the expensive analytical instruments required for detailed molecular experimentation were essentially nonexistent. But in the relatively affluent countries of the Western World, science now follows a different track (except, perhaps, for such aspects of physics and cosmology as “Inflation Theory”), codified by the old maxim: “*Ideas are cheap—what costs in science is are the firm facts needed to prove them right!*” Still, even in the West, it was not until the early 1950s, three decades after Oparin unveiled his solution, that firm-supporting data began to accrue, a breakthrough event that marked the beginning of the modern experimentalist approach.

## 4. Stanley Miller Provides the Evidence

**Figure 6:**
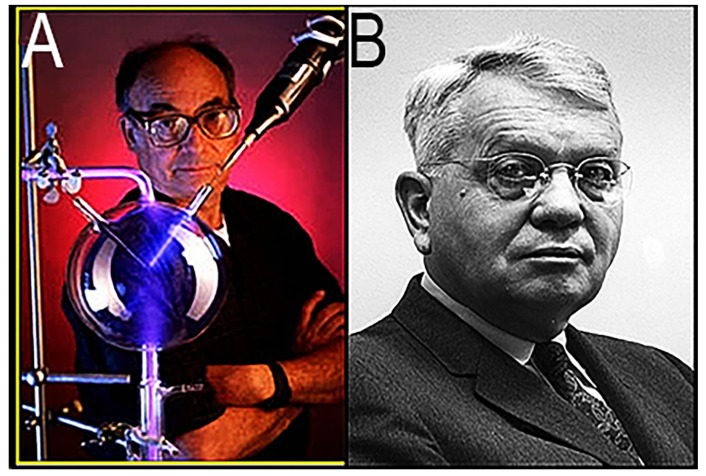
(**A**) Stanley Lloyd Miller. (**B**) Harold Clayton Urey.

**Figure 7:**
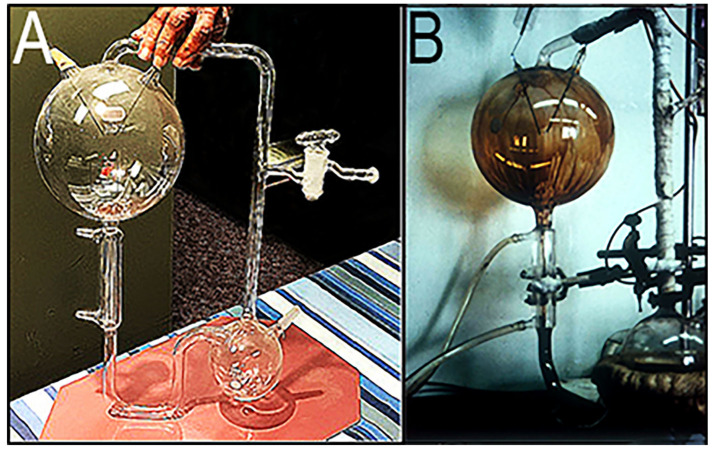
(**A**) Replica of original glassware used in the 1953 Miller–Urey Experiment. (**B**) Miller–Urey experiment after 24 h.

This breakthrough, the famous “Miller–Urey Experiment” of 1953, was carried out by Stanley L. Miller (1930–2007; Figure 6A) while he was a graduate student at the University of Chicago studying under the tutelage of Professor Harold C. Urey (1893–1981; Figure 6B), recipient of the 1934 Nobel Prize in Chemistry for his discovery of deuterium (so-called “heavy hydrogen”). Like Oparin, Stanley was another of my ISSOL friends, so when I was teaching a geochemistry course and hit upon the idea of giving the students the organic sludge from a Miller–Urey synthesis as a set of organic “unknowns” for them to analyze, I called Stanley, then a professor at the University of California, San Diego (as was his former mentor, Harold Urey). Stanley kindly agreed to permit me to borrow his original apparatus to prepare a duplicate. From UCLA, I journeyed down to San Diego, borrowed the glassware, had the replica prepared (Figure 7A), and then returned the original (which is now housed at the Smithsonian Institution in Washington, DC, USA).

The following is the history of Miller’s landmark experiment, recounted to me when I borrowed the original apparatus to have the replica made. Stanley entered the doctoral program at the University of Chicago in September of 1951, initially working on the synthesis of elements under the guidance of theoretical physicist Edward Teller (1908–2003), the famous “father of the hydrogen bomb”. After a year of unproductive work, in the fall of 1952 Stanley listened intently to a presentation by Professor Urey as he postulated that Earth’s primordial atmosphere was almost certainly hydrogen-rich which, powered by the energy of solar UV light, would have led to the production of prebiotic organic molecules that would have rained into the primordial ocean. Inspired by Urey’s talk and regarding this to be a decidedly more promising PhD problem, Stanley visited Prof. Urey and expressed interest in performing the relevant experiments. Urey agreed, but only reluctantly, indicating that he would give Stanley three months to produce results—but that if no results were forthcoming, Stanley would be assigned to investigate thallium in meteorites (a prospect in which Stanley had not the slightest interest).

**Figure 8:**
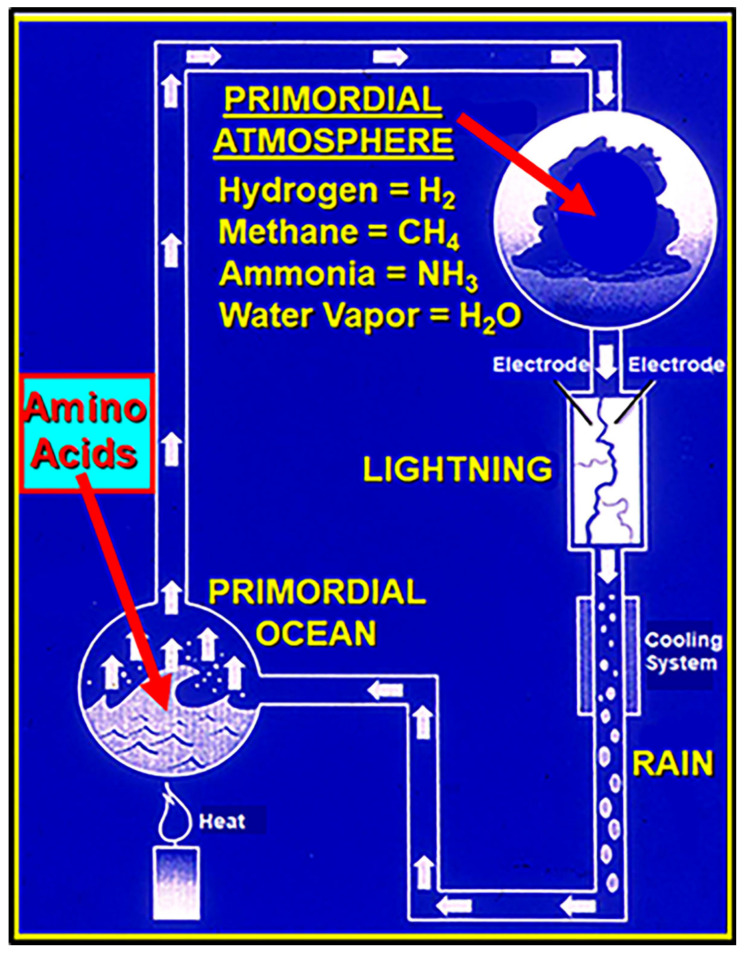
Schematic depiction of the updated apparatus used for the Miller–Urey experiment (with repositioned electrodes) summarizing the basics of the experiment.

Prof. Urey then departed on a three-week lecture tour, and Stanley got to work. He had hoped to use UV as the energy source to power the necessary chemical reactions, but, as shown in Figure 8, because the instrument shop did not have the required quartz glassware, Stanley decided to use the electric discharge to simulate lightning in the “primordial atmosphere”. To this, he added a heat source to continuously reflux the mixture through the apparatus and a cooling system to condense the products into a fine mist that would feed into the “primordial ocean”. Within a week, the apparatus was up and running. As shown in Figure 8, using a hydrogen-rich gas mixture of methane, ammonia, water vapor, and hydrogen gas—the mixture postulated in Urey’s lecture—after only 24 h he observed a dark brown sludge accumulating on the inner surface of the glass-contained “primordial atmosphere” (Figure 7B) and in the simulated “primordial ocean”.

Stanley then used paper chromatography to analyze the sludge and detected four newly synthesized amino acids: glycine, alanine, aspartic acid, and aminobutyric acid. By the time Prof. Urey returned, Stanley had repeated the experiment several times. Prof. Urey then verified the results, and Stanley prepared a manuscript to submit to *Science*. Neither Stanley nor Prof. Urey were then aware of the publications of Oparin and Haldane who decades earlier had proposed similar results using the same starting mixture, papers that were brought to their attention by a reviewer of the submitted manuscript [6].

The reaction to Miller’s 1953 paper was immediate and profound. Scientists worldwide repeated the experiment, confirmed its findings, and set out to extend this new promising approach to unraveling life’s beginnings. Though the Miller–Urey experiment was not intended to create a living organism—not even an archaic, primitive, primordial single cell—it was so enthusiastically trumpeted in the press that soon after its announcement, a U.S. nationwide Gallup Poll posed the question “*Is it possible to create life in a test tube*?” (9% answered yes; 78%, no; and 13% were undecided). Since those days, given the empirical support and enormous impetus provided by the Miller–Urey experiment, the “primordial soup theory” (the Oparin–Haldane hypothesis) has become the foundation of the experiment-based study of abiogenesis, the origin of life from non-biological precursors.

## 5. In Science, Expect the Unexpected

I now depart, briefly, from the historical aspects of the origin of life studies to relate a vignette that illustrates the “fun side” of this science—a topic often neglected in scientific papers but crucial to attracting younger workers into the field—a meeting between A. I. Oparin and Salvador Dalí, the Pioneering Origin of Life Realist meeting the Flamboyant, Creative, Artistic Surrealist.

**Figure 9:**
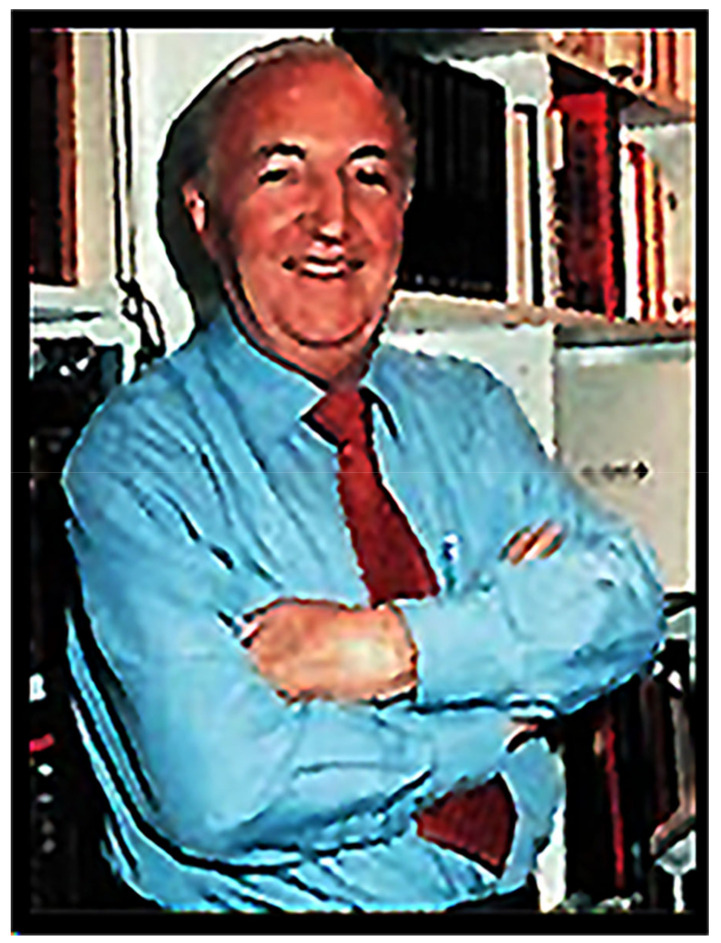
Joan Oró i Florensa.

The triennial 1973 ISSOL meeting was held in Barcelona, the bustling cosmopolitan capital of Spain’s northeastern Catalonia region, well known for the vistas and beach resorts of Costa Brava and, at the far north, the towering Pyrenees Mountains. The chief organizer of the meeting was Joan Oró (1923–2004; Figure 9), Professor of Biochemistry at the University of Houston, Texas. Among Oró’s numerous contributions to the origin of life studies are his prebiotic syntheses of the nucleic acid base adenine from hydrogen cyanide (HCN), a major product of Miller–Urey syntheses [7]. Achieved during the late 1950s, this work stands out as, among the early fundamental results of prebiotic chemistry spurred by the Miller–Urey experiment, important because it initiated research that led to the complete synthesis of the components of gene-defining nucleic acids. Thus, and perhaps even more importantly, Oró’s adenine synthesis paved the way for molecular biologist Alexander Rich (1924–2015) to suggest in 1962 that RNA (ribonucleic acid) could have been the primary genetic material in early life forms, an idea codified in 1986 as the “RNA World” by Harvard biochemist Walter Gilbert. As you will see in the following articles in *Origin of Life 2024*, this concept, the idea that life may have begun with self-replicating RNA molecules rather than the DNA-protein system of modern organisms—in essence, the RNA performing the functions both of genetic material and of enzymes—provides the underpinning of much of the current research in the origin of life field.

As you will see in the following articles in *Origin of Life 2024*, the “RNA World” concept, the idea that life may have begun with self-replicating RNA molecules rather than the DNA-protein system of modern organisms—the RNA thus performing the functions both of genetic material and of enzymes—provides the underpinning of much of the current research in the origin of life field. But not all workers agree that of the various possibilities, RNA holds the answer, a few even harking back to the previously favored concept that the abiotic synthesis of proteins was primary. The same holds true regarding Darwin’s postulated “warm little pond”, currently favored primordial habitats for life’s beginnings ranging from sporadically submerged mudflats to ponds or coastal lagoons, to the open ocean, to submarine volcanic vents, or to a combination of some or all the entire mélanges.

In essence, the origin of life is a complex, multicomponent tangle of seemingly plausible possibilities. Because of this, I have elected to omit from this essay the details of the 1960-to-present Rich–Gilbert–Crick–Orgel et al. “RNA World” breakthrough findings of the origin of life experimentalists, my notion being that because the contributors to *Origin of Life 2024* will deal with such matters in various, perhaps disparate ways, I should in no way prejudice the discussion. In other words, my aim is to set the stage for this in-depth discussion of prebiotic chemistry without me choosing sides/expressing preferences; my views on the current status of that aspect of the field are unimportant; it is the experts, the prebiotic chemists/physicists, who matter. At the same time, I have no doubt that all workers would agree on the significance of the 1859–1960 Darwin–Oparin–Haldane–Miller–Urey–Oró scenario as having laid the foundation for the numerous post-1960 major findings and would credit Joan Oró’s abiotic synthesis of adenine as having played a pivotal role in promoting these advances.

**Figure 10:**
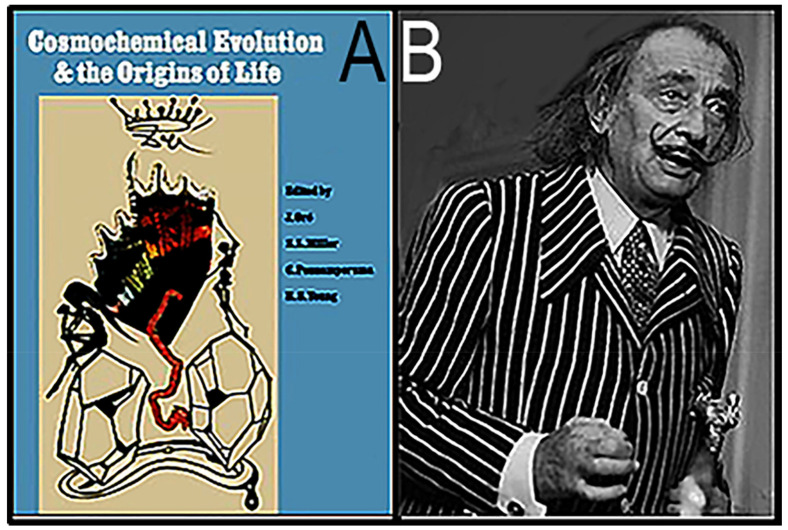
(**A**) The official program for the 1973 ISSOL meeting in Barcelona, Spain, graced by a dreamlike cover illustration prepared by (**B**) Salvador Domingo Felipe Jacinto Dalí i Domènech, Marquess of Dalí.

In addition to being a brilliant origin of life researcher, Oró was also active in Spain’s post-Francoist transition to democracy, serving as an elected member of the Parliament of Catalonia. His role in Catalonian politics provided the contacts that helped him arrange for the 1973 ISSOL meeting to be held in Barcelona, with the cover illustration for the meeting program (Figure 10A) prepared by Oró’s good friend, the surrealist artist Salvador Dalí (1904–1989; Figure 10B).

Although Dalí did not attend the meeting, he wanted to meet Oparin. So, immediately following the confab, he hosted Oparin, Oró and a dozen or so other ISSOLians—me included—at his home in Girona, a several-hour drive northeast of Barcelona on the Costa Brava.

**Figure 11:**
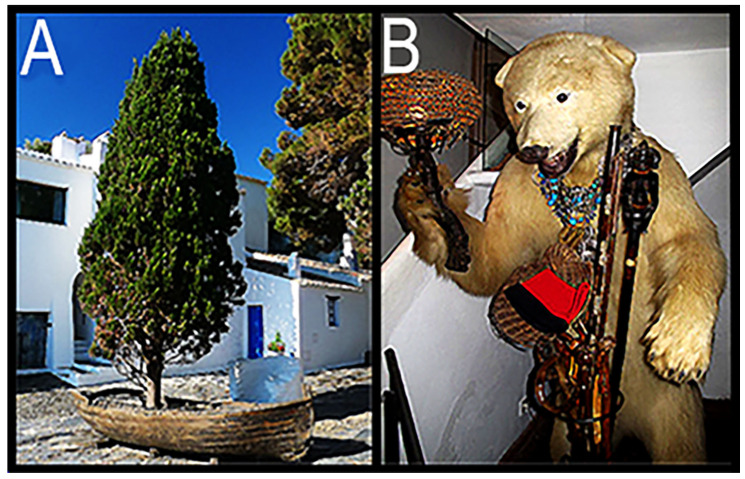
(**A**) The rowboat–pine tree display that fronts Dalí’s home. (**B**) the stuffed bear in the entryway that today welcomes visitors.

Upon our arrival, I was struck by the presence of a rather rickety weathered rowboat—with a tall pine tree arising from its center floorboards (Figure 11A)—beached on a bed of gravel in front of Dalí’s home. “Kinda odd”, I thought.

Our group then ventured up to the front door, where we were greeted by a uniformed, coiffured, rather matronly maid who told us that the señor was awaiting us in the garden. Towering immediately behind her was a large, stuffed brown bear, festooned with an impressive assortment of the señor’s medals and medallions (cf. Figure 11B). I immediately unslung my camera to take a photo, but the maid then held my arm and told me politely but firmly that no photos were permitted in Señor Dalí’s home—that should the need arise, the señor’s personal photographer would be available. Disappointed but willing to abide by the “house rules”, I put my camera away. (Note that the beige-furred bear shown in Figure 11B is *not* the one that confronted us in 1973. Rather, it is of newer vintage, installed sometime later (perhaps after Dalí’s death), evidently to placate the hordes of tourists who even to this day are delighted to visit Dalí’s abode).

Unlike the other homes in Girona, Dalí’s residence consists of three adjoining former fishermen’s houses, backed by a pleasant garden area replete with a serene reflecting pool, the houses interlinked by a narrow subterranean tunnel. Again, the presence of the tunnel is “Kinda odd”, but understandable given Dalí’s political history. Although Dalí was an outspoken supporter of the “Spanish-style autocratic fascist” Francisco Franco (1892–1975), over his many years in power, an increasingly large segment of the Spanish public had become displeased with his dictatorial rule. Dalí wanted to appeal to this disenchanted cohort, and he had the answer: he had once been incarcerated by the Franco regime (albeit by mistake and only over a weekend, a scant three days), so he had the tunnel constructed to remind the public that he, too, had been victimized by Franco’s rule.

Because Oparin’s girth was a bit too large for the narrow tunnel, the maid led him upstairs to enter the garden via a more easily traversed route while the remainder of our entourage navigated the subterranean, dimly lit, jail-like passage. We then all gathered in the garden, where we were greeted by canapés, pink champagne, innumerable toasts, and, of course, the señor.

As we wandered around admiring the sights, Oparin, sitting beside Señor Dalí, gestured to me. His efforts to converse with Dalí had failed: Oparin spoke only Russian—whereas Dalí was fluent in English, Spanish, and French but not Russian—leaving me as the only one in our group available to at least potentially bridge the communication gap. Unfortunately, I was by no means adept at Russian, this being a couple of years before my 6-month sojourn to the USSR, when my Russky markedly improved. Still, “Any port in a storm!”—and, prepared or not, I had been designated to be that “port”. Thankfully, their conversation was easily translatable small talk: Oparin: “*Thank you for inviting us … I looked forward to meeting you*”. Dalí: “*Well, I looked forward to meeting you!*” Oparin: “*You are famous”.* Dalí: “*Yes, but you, too, are famous!*” Oparin: “*Your garden is beautiful”.* Dalí: “*I’m pleased that you like it”.* Then, almost immediately, and to my great relief, I was relieved of duty! We were joined by Oparin’s wife, Nina Petrovna, a former English teacher at Moscow State University and an experienced translator.

**Figure 12:**
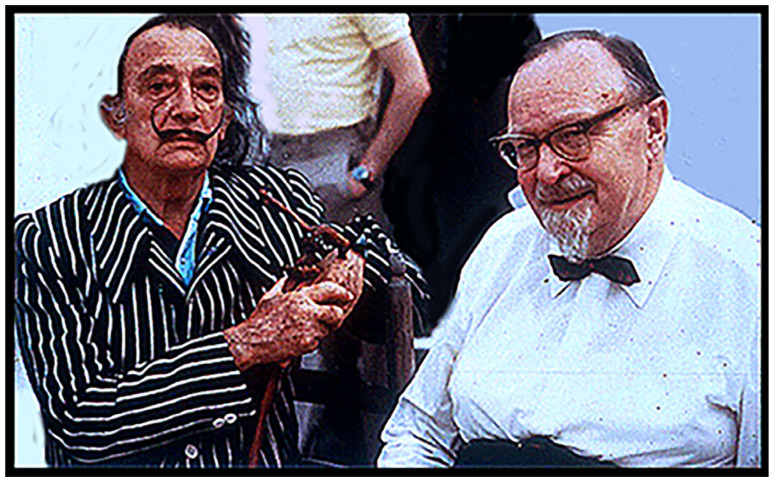
Left to right, Salvador Dalí and Aleksandr Oparin, in Dalí’s garden, Girona, Spain, 1973.

About 20 min later, Oparin looked at me, raised his hands to the front of his face as though he were holding a camera, and said: “*Beeel, vy ne mogli by nas sfotografirovat’, pozhaluysta*?” (“Bill, could please take a photo of us?”). I glanced at Dalí; he nodded his approval, and I took their picture (Figure 12).

**Figure 13:**
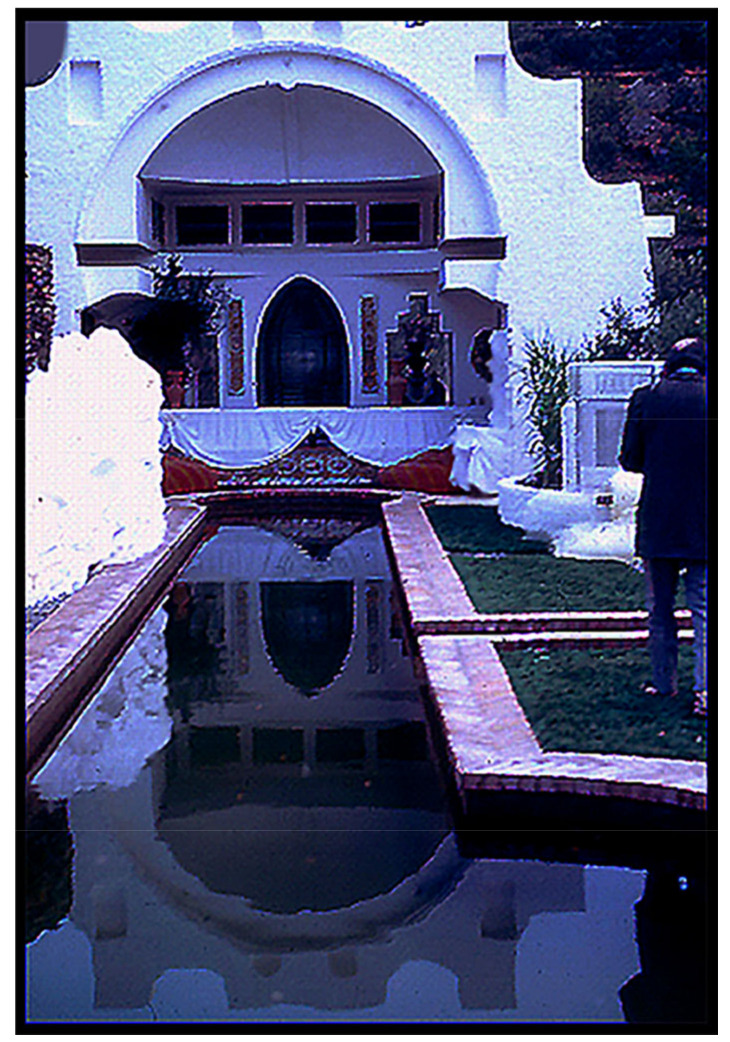
The reflecting pool in the center of Dalí’s garden.

**Figure 14:**
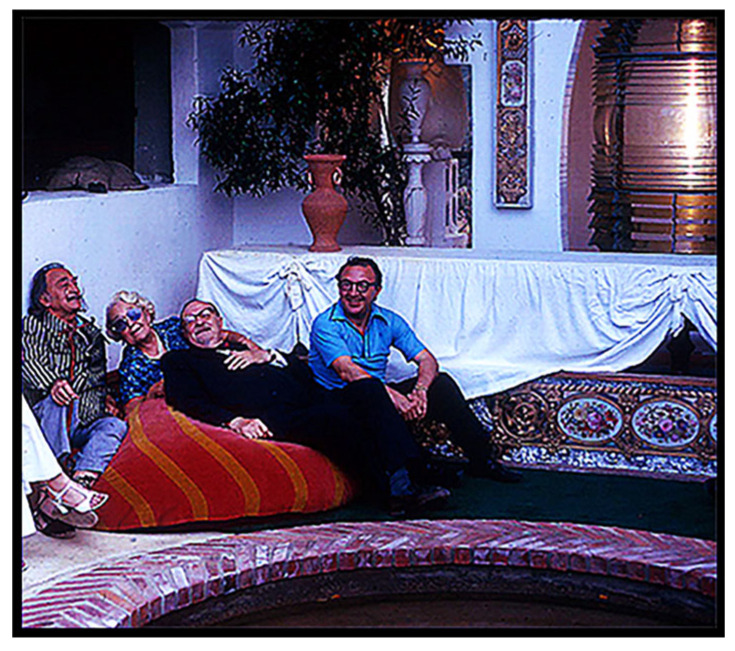
Left to right, Salvador Dalí, Nina Petrovna Oparina, Aleksandr Oparin, and Juan Oro, at the end the reflecting pool adjacent to the lighthouse beacon.

**Figure 15:**
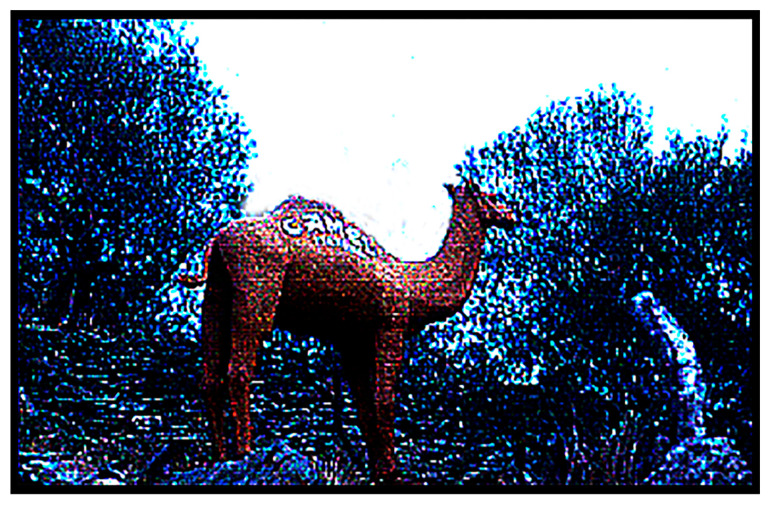
The paper mâché camel on the hillside above the garden, embossed with “Camel Cigarettes”.

**Figure 16:**
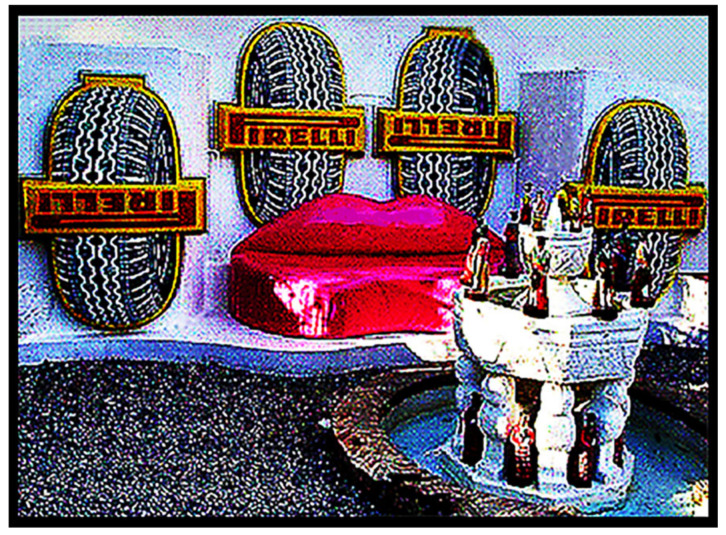
Dalí’s famous loveseat patterned after Mae West’s lips (and backdropped by cardboard cutouts of oversized, upside-down Pirelli tires).

**Figure 17:**
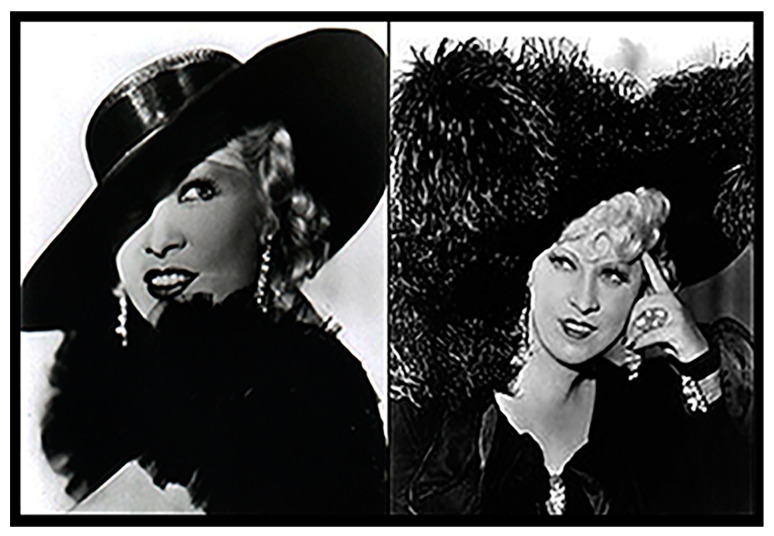
Two photos of Mae West, the actress, singer, and sex symbol inspiration for Dalí’s iconic loveseat.

I had no idea why the señor’s personal photographer was not present to take the picture, but I had now been given “official permission” to take photos, and I did! Figure 13 shows the elongated reflecting pool in the center of the garden, with Dalí on the right, walking on the expanse of bright green plastic grass toward the Persian cupola at its far end that housed a massive lighthouse beacon. Figure 14, taken a bit later, shows the señor and his prime guests, the Oparins and Juan Oró, relaxing next to the lighthouse beacon. Figure 15 depicts Dalí’s paper mâché camel on the hillside immediately above the garden, embossed on its flank with the sales slogan “*Camel Cigarettes*”. And Figure 16 shows perhaps the original version of Dalí’s iconic satin loveseat modeled after Mae West’s lips, Mary Jane “Mae” West (1893–1980) (Figure 17), a world-famous American actress and singer whose career spanned some seven decades. Among her other attributes, she was probably best known as a “sex symbol”, acclaimed for her humorous, bawdy, double-entendres delivered in a husky, low-pitched voice, such as: “*So many men, and so little time!*”; “*Why don’t you come up and see me some time?*”; and “*Is that a gun in your pocket or are you just glad to see me?*”.

Dalí’s splendiferous abode: Fronted by a pine tree-festooned rowboat with a towering bemedaled stuffed bear in its entryway, three houses interlinked by a circuitous jail-like subterranean tunnel leading to a unique back garden replete with an elongated reflecting pool surrounded by plastic grass and centered on a Persian cupula enclosing a massive lighthouse beacon, the garden ornamented by a satin loveseat patterned after Mae West’s lips backed by cardboard cutouts of oversized, upside-down Pirelli tires, and all overseen by a paper mâché camel with “*Camel Cigarettes*” embossed on its hump! Yes, Dalí’s abode was “Kinda odd” … all a product of his bizarre, unfettered, remarkably surrealistic imagination and all *great fun* for us visiting ISSOLians!

## 6. The Solution to Darwin’s Dilemma

**Figure 18:**
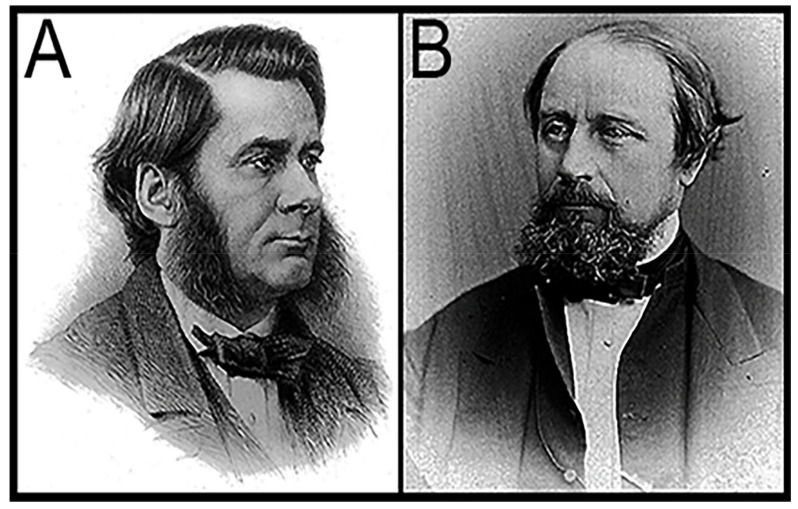
(**A**) Thomas Henry Huxley. (**B**) Sir John William Dawson.

As is recounted at the outset of this essay, Darwin was baffled by the “*inexplicable*” absence of a pre-Cambrian fossil record—evidence of primordial life that might hold the key to understanding how life began—lamenting that this lack of evidence “*may be truly urged as a valid argument against* [the theory of evolution] [1 (Chapter X)]. Though, of course, “absence of evidence is not evidence of absence”—and Darwin had supporters, most prominently Darwin’s “Bulldog” T. H. Huxley (1825–1895; Figure 18A)—naysayers were abundant, many focusing on Darwin’s concern about the “missing” early fossil record.

In North America, probably the most prominent of these strident skeptics was J.W. Dawson (1820–1899; Figure 18B), an anti-evolutionist Scottish Calvinist Presbyterian who, as Principal of McGill University, transformed it into one of Canada’s leading centers of higher education. Dawson was a highly acclaimed academic. He served as President both of the Royal Society of Canada (1882) and the Geological Society of America (1895) and was knighted in 1884 by Queen Victoria (an honor intentionally denied Darwin because of his “anti-religious” writings).

In 1864, only five years after the publication of Darwin’s opus, Dawson was given a set of rock samples by Sir William Edmond Logan (1798–1875), Director of the Geological Survey of Canada. The rocks were geologically old, acquired by an amateur collector from pre-Cambrian, “Laurentian-age” metamorphosed limestones (now known to be ~1100 Ma) along the Ottawa River, west of Montreal. And they were unique, their thin, regular, green, and white lamination differing distinctly from all other rocks of the region, leading Dawson to interpret them as the fossilized shells of exceptionally ancient foraminiferan protozoans—“forams”, for short—even though they were hundreds of times larger than any fossil or modern protozoans then (or now) known. So impressed was he at this “*remarkable fossil … one of the brightest gems in the scientific crown of the Geological Survey of Canada*” [8] that a year later, in 1865, he formally named the putative fossils *Eozoön Canadense*, the “Dawn Animal of Canada”.

To some, this find might well have been regarded as the solution to Darwin’s dilemma, the seminal “breakthrough discovery” showing that the ancient fossil record was neither “unknown” nor “unknowable”. But not to Dawson. Instead, adhering to his staunch Calvinist belief system, he opined: “*There is no link whatever in geological fact to connect Eozoön to the* [diverse fossils] *of the succeeding* [Cambrian and younger geologic record] … *all stand before us as distinct* [Biblical] *creations. Eozoön thus bears damaging negative testimony against evolution* [which] *is incapable of proof and contrary to fact. Evolutionists are mere dreamers, having no scientific basis for their dogma”* [8].

Regardless of Dawson’s religious explication, given that his “find” was in conflict with the widely held dogma that the pre-Cambrian fossil record was nonexistent, it is not unsurprisingly that the scientific community reacted swiftly and negatively to his claim that *Eozoön* was a bona fide fossil.

The first among the numerous doubters were the Irish geologist–mineralogists William King (1809–1886) and Thomas Henry Rowney (1817–1893) who in 1866, only a year after the announcement of the discovery of *Eozoön*, evaluated it as “*purely mineralic, nonbiologic*” [9]. Then, in 1879, a bit more than a decade later, their evaluation was reinforced by Karl Möbius (1825–1908), Professor of Geology at the University of Kiel and the foremost foram specialist in Germany, who examined some 90 sliced specimens of *Eozoön* provided by Dawson and concluded that they were “*certainly nonbiologic*” [10]. And the case was finally sealed in 1894 by Hugh Johnson-Lavis (1856–1914) and John Walter Gregory (1864–1932), geologists at London’s British Museum (Natural History) who discovered blocks of *Eozoön* ejected from Monte Somma, an integral part of the Somma–Vesuvius volcanic complex of southern Italy, and identified the ejecta as metamorphosed, serpentinized limestone, specimens that were assuredly inorganic, nonbiologic, and non-fossil [11].

As the decades-long *Eozoön* saga well illustrates, in the mid- to late-1800s any such claims of the discovery of pre-Cambrian fossils were destined to be controversial and virtually assured to elicit doubt and skepticism. After all, ever since Darwin “everyone knew” that the truly ancient fossil record was both “unknown and unknowable”.

Nevertheless, and although Darwin was correct in stating that in 1859 the pre-Cambrian record of life was entirely unknown, not all paleontologists viewed it as necessarily unknowable. Thus, and not surprisingly, during the early- to mid-1900s, numerous paleontologists, perhaps most prominently Harvard’s trilobite specialist Percy Edward Raymond (1879–1952), set out to solve the problem [12]. But all failed, primarily because they asked the wrong question. By seeking evidence of pre-Cambrian animal fossils similar to those entombed in immediately overlying Cambrian strata, they failed to search for remnants of less advanced, earlier-evolved organisms—such evolutionary precursors as algae, fungi, and their more primitive bacterial ancestors. Moreover, being paleontologists rather than paleobotanists, they may not have realized that, like higher plants, all such earlier evolved life forms had robust cell walls that would be readily preservable—as they are, for example, in petrified woods (by a process more properly known as “permineralization” rather than “petrifaction”).

In retrospect, it thus seems plausible that the problem more properly belonged in the purview of paleobotany rather than paleontology. Yet this, too, would probably have failed, given the temporal separation between the close of the pre-Cambrian and the origin and evolution of higher plants, the prime interest of the paleobotanical community. Indeed, in 1931, the arguably most influential paleobotanist of the day, Sir Albert Charles Seward (1863–1941), effectively scuttled any such approach, asserting that “*We can hardly expect to find in pre-Cambrian rocks any actual proof of the existence of bacteria*” [13].

**Figure 19:**
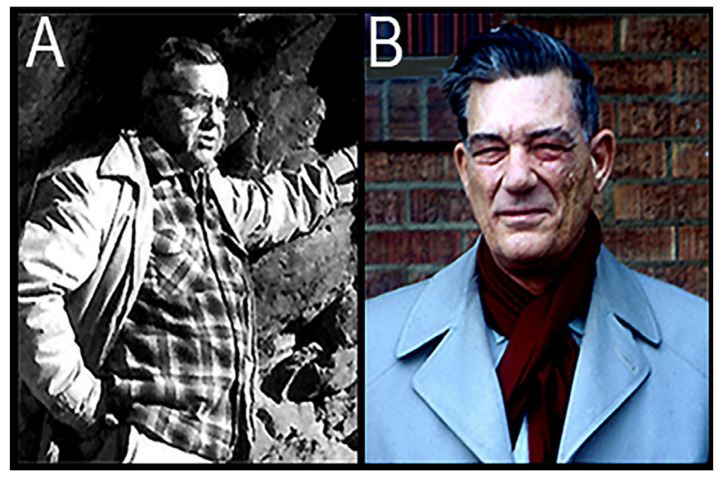
(**A**) Stanley Allen Tyler. (**B**) Elso Sterrenberg Barghoorn.

Thus, by the mid-1900s, the dilemma posed by the “missing” pre-Cambrian fossil record—broached by Darwin in 1859—had remained mired in limbo for a full century. Then, at long last, in 1954, a breakthrough discovery was announced [14], made by University of Wisconsin economic geologist Stanley Tyler (1906–1963; Figure 19A)—not a paleontologist, not a paleobotanist—who had linked up with Harvard paleobotanist Elso Barghoorn (1915–1984; Figure 19B). As might have been predicted, both their announcement paper and, a decade later, their follow-up report in 1965 [15] detailing the occurrence of diverse microscopic fossils in the ~1900 Ma Gunflint Chert of southern Ontario, Canada, met with widespread doubt and skepticism. After all, the skeptics averred, the “so-called fossils” are “far too old”, are for the most part “unlike any fossils previously known”, and represent but a single example isolated in time—“they must be modern contaminants”.

But soon thereafter, from the mid-1960s to the late-1970s, a flurry of new finds was reported—from Australia [16,17,18,19,20,21], South Africa [22], the United States [23,24,25], Canada [26], India [27], France [28] and seven deposits of the Soviet Union [29,30]. The sheer volume of such reports and the similarity of many of the reported fossils to microorganisms living today clinched the case, the details of which have been recently reviewed [31,32] and need not be reiterated here. Suffice it to note that these advances, coupled with the publication in 1983 and 1992 of two massive compendia [33,34] summarizing the then-current status of Precambrian studies, adding new finds and new techniques, and highlighting pressing unsolved problems—prepared by a team of 42 scientists from eight countries (Australia, Canada, Denmark, Germany, South Africa, Sweden, US, and USSR)—resulted in the establishment of a new interdisciplinary international field of science, Precambrian paleobiology, that has attracted hundreds of workers worldwide.

This then brings us to the final topic to be addressed in this opening discussion of *Origin of Life 2024*, namely, when did life begin?

## 7. When Did Life Begin?

There is only one repository known by which to meaningfully address this question, the geological record, which, in principle, might contain direct evidence of a pre-life “primordial soup” and perhaps even remnants of the earliest evolved single-celled organisms. Unfortunately, however, it is this same rock record that thwarts such efforts due to the continuous uplift, metamorphism, erosion, sedimentation, and burial of the mineralic materials that make up the solid surface of the Earth, followed by another and yet another such episode, the continuous rock-making and rock-destroying “geological cycle”. As a result, it is accurate (though grammatically suspect) to state that “the older the rock, the greater the chance it no longer exists!” Thus, for example, only a small percentage, on the order of 5% to 8%, of rocks deposited during the Early Precambrian 2500 to 4000 Ma Archean Era are estimated to have survived to the present, and almost no rocks at all are known from the preceding Hadean Era that extends to the ~4500 Ma formation of the planet. Moreover, it is also unfortunately true that “the older rock, the greater the chance it has been metamorphosed”, a particularly disconcerting observation indicating that though the rock in question—and the paleobiologic evidence it might contain—still exists, its entombed fossils and organic matter may have been so altered by heat and pressure that they are no longer decipherable even by use of the most sensitive analytical techniques.

### 7.1. Detection of the “Primordial Soup”

Although it seems undeniable, as discussed below, that the geological cycle has derailed attempts to firmly document the timing and nature of the origin of life per se, this may not be the case with regard to the existence of the Oparin–Haldane “primordial soup”, a concept widely accepted by the scientific community that has yet to be confirmed by direct, in situ, geological evidence.

As Miller–Urey and similar abiotic syntheses well illustrate, such nonbiological production of organic matter basically requires only an energy source (e.g., lightning and/or UV light), a highly reducing (e.g., CH_4_, H_2_, NH_3_, H_2_0) atmosphere, and the absence of reaction-terminating molecular oxygen (O_2_). Such conditions may have prevailed throughout much of the Archean, hundreds of millions of years after the life-generating phase of Earth’s history, leaving a record in the carbonaceous matter of organic-rich sediments. Indeed, it has long been known that methane (CH_4_), produced by methanogenic bacteria, was present in the Archean environment [35]; that hydrogen (H_2_), generated by the UV-induced photodissociation of water vapor, would also have been present [36]; and that both ammonia (NH_3_), a byproduct of the biological decomposition of organic matter, and water vapor (H_2_0) would have been available as well.

This leaves only abiotic synthesis-inhibiting oxygen (O_2_) to be accounted for, a gas that prior to the origin of oxygen-producing cyanobacteria would have been generated in only trace amounts by the UV-instigated photodissociation of carbon dioxide (CO_2_) and water vapor (H_2_O). Based on phylogenetic analyses of 16S and 23S rRNA (ribosomal ribonucleic acid), the current best estimate of the time of origin of oxygenic cyanobacteria is ~2700 Ma ago [37], a date that fits well with the firmly established ~2400 to ~2100 Ma mass-independent sulfur isotope fractionation-based timing of the cyanobacterially generated so-called “Great Oxidation Event” [38,39,40,41,42]. Thus, and despite the uncertainty inherent in the phylogenetically estimated time of origin of oxygen-producing cyanobacteria, it seems reasonable to assume that abiotic organic syntheses could have occurred throughout much or perhaps all of the Archean Era.

If such is the case, why is it that products of nonbiological syntheses have yet to be detected in the carbonaceous matter preserved in Archean sedimentary rocks? The answer is simple: any such abiotic organics would be such a minor component of the preserved carbonaceous material that they would be virtually undetectable, overwhelmed by co-deposited and far more abundant biologically produced organic matter. Sedimentary carbonaceous matter—that of coal, black shales, and black cherts, for example, and properly known as “kerogen”—is derived from the buried, partially decayed, preserved remnants of previously living systems. Once life had originated and proliferated, the biota became a far more voluminous producer of organics than relatively inefficient abiotic syntheses. Thus, even if abiotic organic matter were to be present in the sedimentary rock record throughout much of the Archean, it would be “swamped out” at a ratio of many thousands to one by the organics produced by life.

Given this and the virtual absence of a pre-4000 Ma Hadean rock record, the repository that would be expected to harbor evidence of the pre-life stage in Earth’s development, the search for remnants of the primordial soup devolves into a proverbial “needle in a haystack” hunt. But success is not out of the question. Stemming from the seminal studies of Philip Hauge Abelson (1913–2004) and Thomas C. Hoering (1925–1995) [43], the preservation in bulk samples of relatively unaltered sedimentary kerogens of the stable isotopes of carbon, ^12^C and ^13^C, has been long established, their relative abundance being a function of enzyme-mediated mass-dependent kinetic isotopic fractionation (the lighter isotope, ^12^C, moving more rapidly and therefore coming into contact with the catalytic enzyme more frequently than heavier ^13^C). Documented in thousands of such kerogenous Phanerozoic and Precambrian sediments, carbon isotopic ratios have been used to trace the record of biologic photosynthesis, initially anoxic and only later O_2_-producing [32], to at least ~3500 Ma ago [44].

Kerogens, however, are geochemically altered remnants of originally deposited organics, and as they mature toward graphite, their geochemically stable pre-diamond endpoint, their originally disordered substituents become aggregated into the increasingly larger and more ordered plate-like polycyclic aromatic hydrocarbons (PAHs) that make up the graphene layers of maturing kerogens. Thus, in mature kerogens, much of the original enzyme-determined detailed molecular structure has been altered or destroyed. In less mature kerogens, however, this is not the case, and it is here—in the relatively unaltered molecular bridges that link together the constituent PAHs—that evidence of the abiotic soup might be detected.

As is well demonstrated by the pioneering studies of John Michael Hayes (1940–2017) [45], the intramolecular distribution of the stable isotopes both of carbon (^12^C and ^13^C) and of hydrogen (^1^H and ^2^H) in biosynthesized organic matter is, like the isotopic constituents of bulk samples of unmetamorphosed kerogens, a result of enzymatic syntheses with such products having regular, predictable, isotopic distributions. In contrast, the products of Miller–Urey and all other types of abiotic syntheses are characterized by randomly distributed carbon and hydrogen isotopes. Thus, the intramolecular carbon and hydrogen isotopic distributions in the relatively unaltered linkage groups that tie together the PAHs of kerogenous graphene layers appear to provide a promising source of data by which to distinguish biotic and abiotic carbonaceous sedimentary organic matter. Though the abiotic signal can be expected to be minimal, it might well be discernable and, thus, provide telling evidence of the hypothesized non-biological production of organic matter early in Earth’s history.

### 7.2. The Oldest Records of Life

As a result of the “geological cycle”, only a minuscule fragment of the 2500 to 4000 Ma Archean rock record has survived to the present. Moreover, only four terrains older than ~3000 Ma are currently known, listed in the following by their ascending geologic age (which, as might be expected, correlates both with their increasing metamorphic grade and their decreasing areal extent): (1) the 1800 to 3800 Ma, relatively little metamorphosed, ~250,000 km^2^ Pilbara Craton of northwestern Western Australia; (2) the 3500 to 3700 Ma, little to moderately metamorphosed, ~7200 km^2^ Barberton Greenstone Belt of South Africa; (3) the 3700 to 3800 Ma, moderately to highly metamorphosed, ~140 km^2^ Isua Greenstone Belt of southwestern Greenland; and (4) the 3750 to 4280 Ma, typically rather severely metamorphosed, ~20 km^2^ Nuvvuagittuq Greenstone Belt of northern Québec, Canada.

Although possible evidence of life—alleged microfossils and/or putative microbialite stromatolites—have been described from each of these especially ancient terrains, e.g., [46,47], the evidence is slim, except for that known from the Pilbara Craton, and none of the reports pertains to life’s earliest formative stages. Thus, the current contribution of the known fossil record to the origin of life problem is necessarily limited to that of providing both a minimum age for the existence of biological systems and an understanding of how far and fast evolution had proceeded by the time that evidence had become entombed. (As an aside, however, it should also be noted that due to global warming and the melting of the Greenland ice sheet, potentially fossiliferous rock strata will become exposed that could reveal firm evidence of life appreciably older than that now known).

Of the four especially ancient terrains listed above, the least metamorphosed and paleobiologically most studied is the Pilbara Craton, a repository of the best documented and most intensively analyzed ~3500 Ma stromatolites [48] and microfossils now known. Although such Paleoarchean stromatolites provide firm evidence of ancient microbial communities and their microbialite-building capabilities, unless they contain cellularly preserved microorganisms, they cannot reveal the specific types of microorganisms involved. In contrast, the discovery of cellularly preserved fossils can evidence the degree of biotic diversification achieved by that stage in evolutionary development and, thus, the “evolutionary distance” that separates them from life’s beginnings. It is for this reason that we here focus on the oldest assemblage of bona fide microfossils now known, that of the Pilbara Craton’s ~3465 Ma Apex chert.

First reported three decades ago, in 1993 [49], the Paleoarchean Apex fossils have been widely touted as the “oldest evidence of life on Earth”. It is thus not surprising that they have been subject to intense scrutiny, making them the most thoroughly studied Precambrian microfossils in the history of science. As a result, not only has their biogenicity been firmly established, but the biological affinities of several of the Apex taxa have now been established, and similar fossils of nearly the same age have come to light. In short, the Apex fossils have “stood the test of time”.

**Figure 20:**
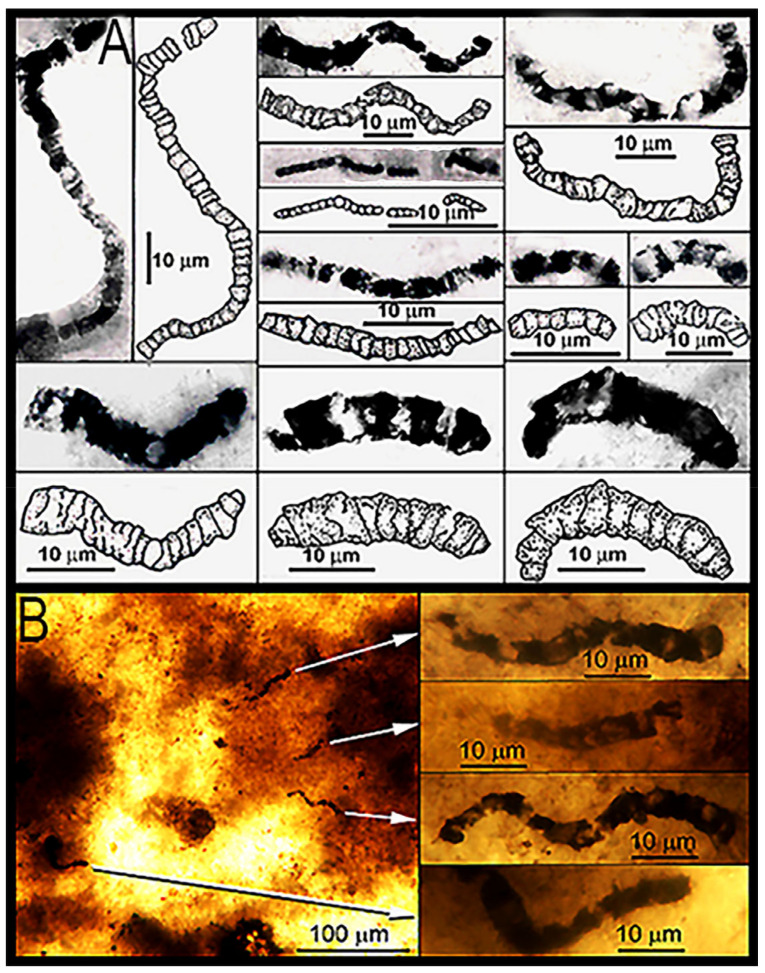
(**A**) The Apex fossils are varied and abundant (left column, top to bottom: *Primaevifilum amoenum*, *P. conicoterminatum*; middle column, top to bottom: *P. amoenum, Archaeotrichion septatum, P. amoenum, P. conicoterminatum*; and right column, top to bottom: *Primaevifilum amoenum*, *P. conicoterminatum*; *P. conicoterminatum*; *P. conicoterminatum*). (**B**) Four rather closely spaced specimens of *Primaevifilum amoenum* in a single focal plane and optical field of view.

**Figure 21:**
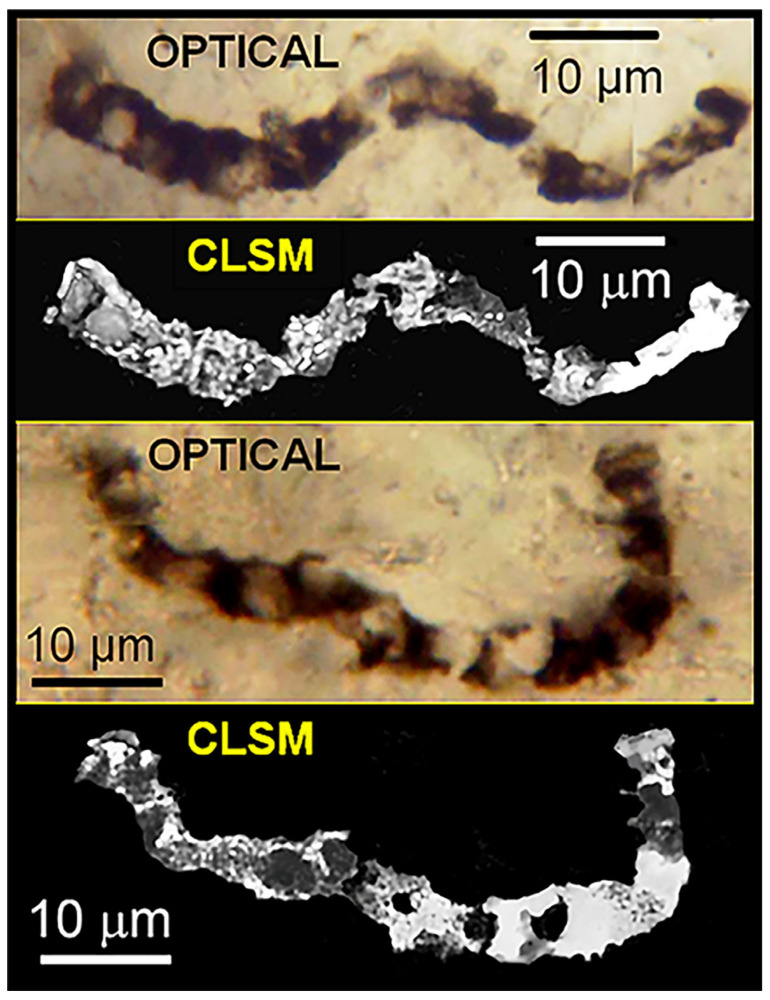
Optical (color) and confocal laser scanning microscope (CLSM) black-and-white images of two specimens of *Primaevifilum amoenum* permineralized in the Apex chert, the CLSM images documenting the presence of carbonaceous matter.

**Figure 22:**
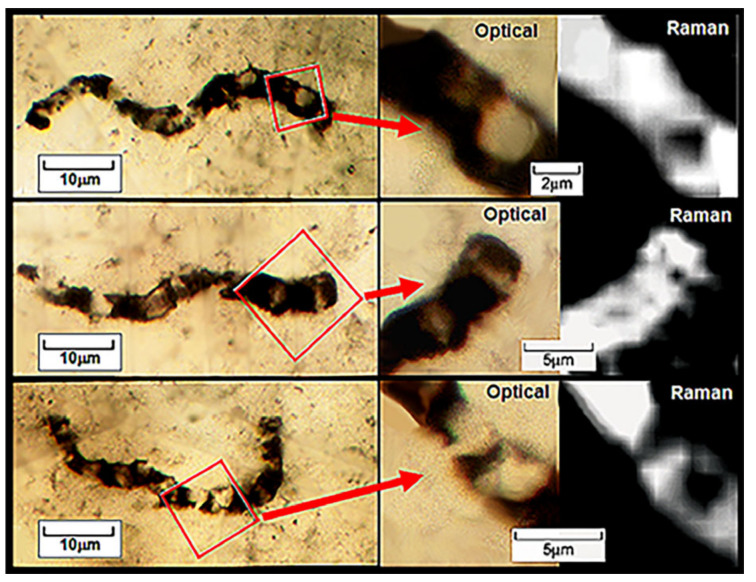
Optical and Raman images of three specimens of *Primaevifilum amoenum* permineralized in the Apex chert, the Raman images recording the “D-Band” of the kerogenous cell walls and, thus, documenting the distribution of carbonaceous matter.

**Figure 23:**
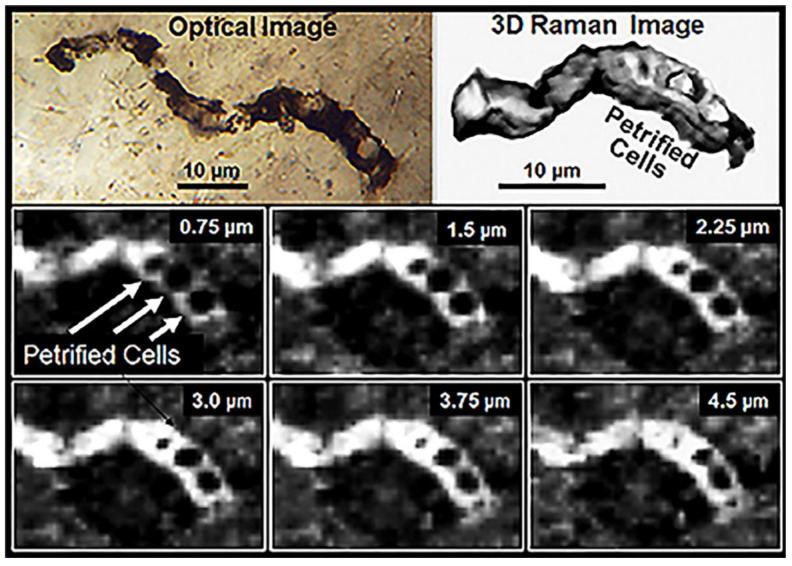
An optical image (upper left) and three-dimensional Raman images of the Raman “D-Band” of a specimen of *Primaevifilum amoenum* permineralized in the Apex chert; the bottom six images are Raman images illustrating progressively deeper slices through the fossil (ranging from 0.75 μm to 4.5 μm below its upper surface), showing that the cell diameters vary with depth and that the cell lumina are devoid of residual cytoplasm.

The evidence is impressive: In the 11 taxa of filamentous, dark brown to black carbonaceous microfossils formally described from the Apex chert [49], 1877 cells have been measured in 180 filaments ranging from 0.5 to 19.5 μm in width and 28 to 89 μm in length. The fossils are demonstrably indigenous to and syngenetic with their embedding chert matrix and, like the filamentous microbes of modern biocoenoses, they are abundant and morphologically varied (Figure 20), are composed of carbonaceous matter (Figure 21, Figure 22 and Figure 23), and are cellular, cylindrical, and unbranched (Figure 20, Figure 21, Figure 22 and Figure 23).

Because the biological affinities of the Apex fossils within the Procaryotae could not be firmly established when they were first reported, they were initially described as prokaryotes incertae sedis [49], i.e., “bacterium-grade microorganisms of uncertain systematic relations”. More recent studies, using secondary ion mass spectrometry (SIMS) to analyze in situ the carbon isotopic composition of individual fossils [50], have clarified the biological relations of several of the Apex taxa, documenting the presence of anoxygenic photosynthetic bacteria (*Primaevifilum minutum*, N = 4, δ^13^C = −30 ± 1), archaeal methanogens (*Primaevifilum delicatulum,* N = 5, δ^13^C = −35 ± 1), and archaeal methanotrophs (*Archaeoscillatoriopis disciformis,* N = 4, δ^13^C = −40 ± 1).

Other recent studies [51] have similarly documented the presence of anoxygenic photosynthetic bacteria in the ~3400 Ma Strelley Pool Formation of the Pilbara Craton, a Paleoarchean deposit only slightly younger than the Apex chert, in which these mat-building, light-requiring, obligately anaerobic photosynthesizers occur in mudflat-like deposits. Their presence, indicative of the essentially anoxic nature of the immediately overlying Paleoarchean atmosphere, is consistent with their intermixed co-occurrence with anaerobic sulfuretum bacteria, sulfate- (SO_4^−^_) and nitrate- (NO_3^−^_) cycling microbial assemblages known also from two Paleoproterozoic sub-seafloor deposits [52,53] that in the modern world inhabit oxygen-deficient muds off the western coasts of South and Central America [54].

**Figure 24:**
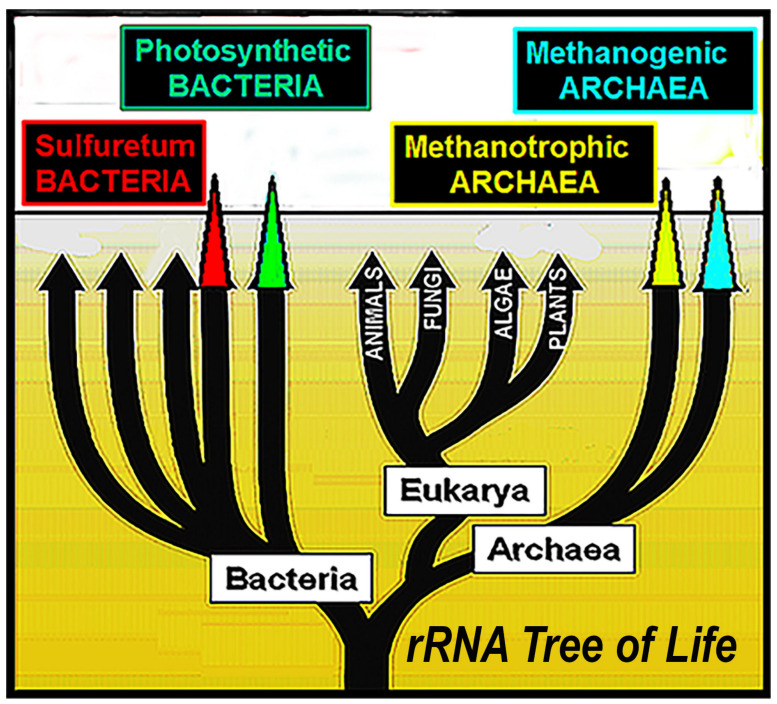
Simplified rRNA tree of life showing that all four of the oldest fossil lineages now known—bacterial sulfuretum microbes, bacterial anoxygenic photosynthesizers, archaeal methanotrophs and archaeal methanogens—are situated near its base.

Thus, the facts are firm: at least four distinct phylogenetic lineages were extant by ~3400 Ma: (1) archaeal methanogens; (2) archaeal methanotrophs; (1) bacterial anoxygenic photosynthesizers; and (4) bacterial sulfate- and nitrate-cycling sulfuretum microbes. Notably, each of these four groups is situated near the base of the rRNA phylogenetic tree of life (Figure 24), not only adding credence to such molecular biology-based broad extrapolations but also firmly indicating that primordial life evolved early, far, and fast.

## 8. Summary and Conclusions

This essay, a broad historical synthesis leading up to the *Origin of Life 2024* studies presented in the remainder of this volume, has reviewed a series of significant contributions made by the origin of life community toward resolving this “greatest unsolved problem of modern science”. The studies discussed track two parallel but interrelated paths, that of the imaginative, creative experimenters and that of the real-world observationalists, both paths dating from Darwin.

For the experimentalists, the story begins with Darwin’s 1871 letter to Joseph Hooker, in which he speculates: “… if (and oh what a big if) we could conceive in some warm little pond with all sorts of ammonia and phosphoric salts,—light, heat, electricity &c. present, that a protein compound was chemically formed, ready to undergo still more complex changes…” This review then continues with a discussion of the development of the Oparin–Haldane “primordial soup” hypothesis (in 1924 and 1929); of the Miller–Urey synthesis of amino acids under primitive Earth conditions (in 1953); and of Joan Oró’s 1959 abiotic production of the nucleic acid adenine, the first major step toward understanding the pre-life origin of genetic material.

For the observationalists, those seeking direct evidence of life’s beginnings, the story begins with Darwin’s 1859 plaintive remonstration in *On the Origin of Species* that “*If the theory* [of evolution] *be true, it is indisputable that before the lowest Cambrian stratum was deposited … the world swarmed with living creatures.* [Yet] *why we do not find rich fossiliferous deposits belonging to these assumed earliest periods … I can give no satisfactory answer. The case at present must remain inexplicable*”. The problem having thus been broached, the observationalists immediately set out to solve Darwin’s dilemma, first evidenced by the rancorous controversy surrounding J.W. Dawson’s “Dawn Animal of Canada” (1864–1894). This episode was then followed by a persistent but unrewarded search for evidence of pre-Cambrian life by Percy Raymond and many others during the early 1900s—efforts stymied by the searchers’ lack of understanding of the problem at hand backed by A.C. Seward’s 1931 unfounded dictum that “*We can hardly expect to find in pre-Cambrian rocks any actual proof of the existence of bacteria*”.

The two-decade hiatus that then resulted was followed by Stanley Tyler’s initially much maligned 1953 “breakthrough find” of Precambrian microfossils in the ~1900 Ma Gunflint chert of Ontario, Canada; an onslaught of new finds worldwide in the 1960s–1980s; and the publication in 1983 and 1992 of two massive monographs that provided firm footing for the establishment of Precambrian paleobiology, a new, interdisciplinary international field of science. Discoveries of even older evidence of life then followed, in 1993 of the oldest microfossil assemblage now known, that of the ~3465 Ma Apex chert of Western Australia and, in 2017, of a second Paleoarchean assemblage, that of Western Australia’s 3400 Ma Dresser Formation, the combined biotas of the two deposits documenting the Paleoarchean existence of four early evolved lineages, archaeal methanogens and methanotrophs, and bacterial anoxygenic photosynthesizers and bacterial sulfur- and nitrogen-cycling sulfuretum microbes, all lineages that are situated near the base of the rRNA phylogenetic tree of life.

Clearly, the origin of life community has made major progress toward resolving the timing and nature of how life began, but much more remains to be learned. Though the real-world observationalists have shown that primordial life evolved early, far, and fast, their efforts to uncover the formative stages of life’s existence have come to naught, thwarted by the “geological cycle” and the paucity of rock strata appreciably older than those already investigated. This, then, leaves the problem in the purview of the imaginative, creative experimentalists, the bulk of the origin of life community. As you will see in the following articles in *Origin of Life 2024*, their work can be expected to hold the answer. Read, learn, ponder, and enjoy—a great triumph awaits those origin-of-lifers who ultimately define and link together the myriad processes and products that led to the emergence of life on Earth, the “greatest of all unsolved problems in modern science”.

## Data Availability

Not applicable.
